# Time-Modulated Local Defect as a Mechanism for Controlled Frequency Conversion in a Finite Phononic Crystal

**DOI:** 10.3390/ma19214652

**Published:** 2026-10-31

**Authors:** Sebastian Garus

**Affiliations:** Faculty of Mechanical Engineering and Computer Science, Department of Mechanics and Fundamentals of Machine Design, Czestochowa University of Technology, Dabrowskiego 73, 42-201 Czestochowa, Poland; sebastian.garus@pcz.pl

**Keywords:** phononic crystal, metamaterials, TMM, FDTD, space–time defect

## Abstract

This work investigates the effect of a dynamically modulated defect on the propagation of mechanical waves in a quasi-one-dimensional phononic crystal. The studied structure consisted of periodically arranged meta-atoms with different cross-sectional areas, and a local defect enabled a periodic change in the geometry of one of the elements. The transmission characteristics of the static structures were determined using the transfer matrix method (TMM), while wave propagation in space and time was analyzed using the finite difference time-domain method (FDTD). Numerical results were experimentally verified using a four-microphone impedance tube. It was shown that changing the defect cross-sectional area allows for controlling the fundamental bandgap width and the positions of adjacent transmission maxima and minima. The introduction of periodic defect modulation led to modulation of the transmitted wave amplitude and the generation of additional spectral components with frequencies $f_{s}+kf_{p}$, related to the periodic time modulation. Increasing the modulation frequency and amplitude resulted in an increase in the observed effects and amplitudes of the generated frequency components. The conducted analysis showed that the dynamic defect can be used to control the transmission and conversion of frequencies in phononic structures.

## 1. Introduction

Phononic crystals (PnCs) and acoustic metamaterials are artificially designed structures in which the propagation of acoustic and mechanical waves can be controlled by the appropriate selection of geometric and material properties. The periodic arrangement of structural elements and their parameters influence the occurrence of frequency ranges in which the transmission of waves is reduced, which is called the formation of a band gap. These phenomena are related to Bragg scattering, local resonances, or the simultaneous occurrence of both mechanisms [1,2,3,4]. The position and width of the band gaps depend on the geometry, material properties and spatial arrangement of the structural elements. Even simple one-dimensional structures composed of elements with alternating cross-sections or acoustic impedances can exhibit selective wave transmission, which makes it possible to design devices such as acoustic filters, vibration isolators and waveguides [3,5,6,7,8].

The introduction of a local defect into a phononic crystal causes a disturbance of its periodicity, which can lead to the formation of additional transmission states within the band gap, the localization of acoustic energy, and the occurrence of resonant phenomena characteristic of local resonators or acoustic microcavities [7,9,10]. By changing the geometric and material parameters of the defect, such as its length, thickness, cross-section, or acoustic impedance, it is possible to control the resonant frequencies and modify the transmission bandwidth and maximum transmission value [11,12]. The conducted analysis of such structures makes it possible to determine how a local modification influences the transmission characteristics of the entire finite structure, while the periodic arrangement of the remaining unit cells is preserved.

Classical phononic crystals and metamaterials are essentially static after fabrication, and modifying their transmission characteristics requires geometric changes, tunable materials, or additional actuators. Time-dependent structural parameters provide an alternative means of controlling wave propagation and enable effects unavailable in static structures [13].

Another approach is based on the periodic temporal modulation of material or geometric parameters of the structure [14]. If the selected structural parameter changes periodically with a modulation frequency $f_{p}$, the interaction of a wave with a source frequency $f_{s}$ with the dynamic structure can result in the formation of additional frequency components. In the simplest case, the frequencies of the resulting components can be described by the following relationship:

(1) $$f_{k}=f_{s}+kf_{p},\;k=0,\;\pm 1,\;\pm 2,\;\ldots$$

where $f_{k}$ represents the frequency of the *k*-th component, $f_{s}$ is the frequency of the source wave and $f_{p}$ is the modulation frequency. The obtained frequency components are referred to as Floquet harmonics or sideband frequency components. Their formation is a result of the periodic modulation of the system parameters and causes the energy of the wave, initially concentrated at a single frequency, to be distributed among several discrete frequency components.

Periodic temporal modulation significantly changes the propagation of waves in the structure. In a static structure, the frequency of the propagating wave remains essentially unchanged, while in a time-modulated structure it is possible to couple different frequency components. When the modulation occurs simultaneously in space and time, the structure is called a space–time-modulated structure [15]. Such structures make it possible to control the spatial and temporal properties of wave propagation independently or in a coupled manner, which allows for the occurrence of phenomena such as frequency conversion, active control of wave propagation, parametric amplification and non-reciprocal transmission [14]. This phenomenon is also observed for photonic metamaterials [16].

The phenomenon of frequency conversion occurs as a result of periodic temporal modulation. In this case, the energy of the wave with the frequency $f_{s}$ is transferred to components with frequencies $f_{s}\pm f_{p}$, $f_{s}\pm 2f_{p}$, $f_{s}\pm 3f_{p}$, and so on [17]. The modulation frequency determines the distance between the successive components, while the modulation amplitude affects the intensity of the coupling between them. An increase in the modulation depth results in higher amplitudes of the generated harmonics and increases the transfer of energy from the fundamental frequency to the sideband components. The conducted analysis shows that changing the modulation parameters makes it possible to control the transmission characteristics of the wave.

This phenomenon is important for the design of active acoustic systems. Unlike classical filters, whose characteristics are fixed during the design process, time-modulated structures make it possible to change the response during operation [13]. By using such structures, it is possible to design tunable filters, frequency converters, systems for active control of wave propagation, and structures with reconfigurable spectral properties. In the works on spatio-temporal structures, it has been shown that the appropriate selection of the modulation frequency and amplitude makes it possible to control the position and amplitude of the generated spectral components [14,16,18].

Several works have focused on controlling the propagation of acoustic waves by using temporal and spatio-temporal modulation. In [13], Wen et al. experimentally demonstrated spatio-temporal effective acoustic media using meta-atoms with dynamically switched resonance strengths, which made it possible to describe metamaterials with time-dependent effective parameters. In the work [19] the authors demonstrated unidirectional amplification and enhanced frequency conversion in a non-Hermitian spatio-temporally modulated acoustic metamaterial. The obtained results were achieved by controlling the temporal modulation of gain and loss and introducing a phase delay between adjacent meta-atoms. In [16] the authors analyzed frequency conversion and nonreciprocal transmission in a reconfigurable space–time-modulated metamaterial based on a field-focused cavity, in which temporal modulation was combined with spatial field control. More recently, in [14] the authors demonstrated parametric amplification and frequency conversion in a one-dimensional acoustic metamaterial with space–time-modulated effective density. The conducted research showed that temporal modulation can be used to control the spectral and directional properties of acoustic waves.

The analysis of acoustic wave propagation in spatio-temporally modulated structures has focused on different mechanisms and objectives, such as the realization of effective spatio-temporal media, non-Hermitian amplification, nonreciprocal transmission and parametric frequency conversion. However, the influence of periodic modulation confined to a single mechanically actuated defect on wave propagation and the spatial distribution of frequency components in a finite phononic crystal with a prescribed static band structure has not been established in these works. The analysis makes it possible to determine how a localized modulation mechanism influences the spectral properties of propagating waves without changing the geometry of the surrounding periodic structure.

In this work, temporal modulation is applied to a single defect, whose effective cross-sectional area changes periodically due to the motion of a piston. The equilibrium position of the piston determines the static geometry of the defect and thus the baseline transmission characteristics, while the modulation frequency and amplitude determine the time-dependent response of the structure. The proposed configuration makes it possible to combine transmission control resulting from the geometry of the defect with frequency conversion induced by modulation in a finite phononic crystal, without the need to modulate all unit cells in time. The aim of this work is to determine how a single local geometric modulation influences the coupling between spectral components and their propagation through a finite structure.

The investigation further combines transfer matrix analysis of the static transmission response, finite-difference time-domain simulations of the time-dependent acoustic field, and experimental measurements using a four-microphone impedance tube. Particular attention is paid to the spatial distribution of the generated frequency components, in addition to their amplitudes in the transmitted spectrum. The effects of the equilibrium defect geometry, modulation frequency, and modulation amplitude are examined to determine how these parameters influence the static response and the dynamic frequency-conversion process. This approach provides a basis for assessing the potential of a locally modulated defect as a means of reconfigurable spectral control in finite phononic structures.

The purpose of the conducted research was to analyze the influence of a single time-modulated defect on wave transmission and frequency conversion in a finite phononic crystal. The influence of the equilibrium piston position L, the modulation frequency $f_{p}$ and the modulation amplitude $A_{p}$ was investigated. The equilibrium piston position determined the static defect geometry, the modulation frequency controlled the spectral spacing of the generated Floquet harmonics, and the modulation amplitude determined the extent of the periodic geometric variation. The analysis of the transmission characteristics and the spatial distribution of the generated frequency components was carried out numerically and experimentally.

The static transmission characteristics of the structure were analyzed using the Transfer Matrix Method (TMM), which enables the identification of band gaps, transmission maxima, and the influence of defect geometry [20]. Wave propagation and the effects of temporal modulation were investigated using the Finite-Difference Time-Domain (FDTD) method, which accounts for the time-dependent acoustic field and spectral changes [15,21]. The numerical results were experimentally verified using a four-microphone impedance tube in accordance with the measurement approach described in ASTM E2611 [22,23]. The experimental validation also accounts for imperfections and losses that may not be fully represented in numerical models.

The aim of the work was to determine the influence of the periodic change of the local defect cross-section on transmission and frequency conversion in a finite quasi-one-dimensional phononic crystal. The analyzed structure consists of periodically arranged elements with different cross-sections and a local defect, whose effective cross-section can be changed by the movement of a piston. In the first stage, the influence of the static position of the defect on the transmission characteristics was determined using the Transfer Matrix Method (TMM). Subsequently, the Finite-Difference Time-Domain (FDTD) method was used to analyze wave propagation in the finite structure and the influence of periodic modulation of the defect on the acoustic field and wave spectrum. Particular attention was paid to the generation of additional frequency components described by the relation $f_{k}=f_{s}+kf_{p}$, as well as their amplitudes and spatial distribution in the structure. Finally, the numerical results were verified experimentally using a four-microphone impedance tube.

The analysis was carried out for three parameters of the dynamic defect. The first parameter is the equilibrium position *L*, which determines the cross-section of the defect and its static transmission characteristics. The second parameter is the modulation frequency $f_{p}$, which determines the distance between the successive Floquet harmonics. The third parameter is the modulation amplitude $A_{p}$, which determines the change in the defect cross-section and affects the amplitude of the generated components. The influence of these parameters on the transmission characteristics and frequency conversion was analyzed.

The formulated research problem makes it possible to analyze a locally modulated defect as a mechanism that combines the shaping of transmission characteristics by the geometry with spectral control by temporal modulation. The obtained results make it possible to determine whether a local periodic change in the geometry of a single element can lead to controlled frequency conversion in a finite phononic crystal and how the modulation frequency and amplitude affect this process. This approach can be used as a basis for the development of reconfigurable phononic structures with tunable transmission characteristics, in which the filtering and frequency conversion properties can be changed without modifying the entire structure.

## 2. Materials and Methods

The transmission of static structures was determined using the TMM algorithm. Acoustic wave propagation was analyzed using the FDTD algorithm for both static and dynamic structures. The structures analyzed in this work were made from meta-atoms produced using 3D printing technology. The acoustic properties of the resulting structures were determined using an impedance tube.

### 2.1. Transfer Matrix Method Algorithm

In quasi-one-dimensional structures, the acoustic wave in element *j* is a superposition of waves traveling to the right and left

(2) $$p_{j}=A_{j}e^{-ik_{j}x}+B_{j}e^{ik_{j}x},$$

where $p_{j}$ is acoustic pressure, $A_{j}$, $B_{j}$ are respectively the amplitudes of the waves traveling to the right and left and ω=2πf is angular frequency for frequency *f*.

The effective acoustic impedance is described as

(3) $$Z_{\mathrm{eff},j}=\frac{S_{j}}{c_{0}},$$

where $c_{0}$ is sound velocity in the air and $\rho_{j}$ is the effective mass density defined by

(4) $$\rho_{\mathrm{eff},j}=\frac{S_{j}}{c_{0}^{2}}.$$

These parameters are effective quantities derived from the one-dimensional wave equation for a corrugated tube waveguide with a spatially varying cross-sectional area [24]. They are defined as $\rho_{\mathrm{eff},j}=S_{j}/c_{0}^{2}$ and $K_{\mathrm{eff},j}=S_{j}$, where $S_{j}$ is the cross-sectional area of element *j* and $c_{0}$ is the sound velocity in air. The corresponding effective impedance parameter is $Z_{\mathrm{eff},j}=\sqrt{\rho_{\mathrm{eff},j}K_{\mathrm{eff},j}}=S_{j}/c_{0}$. These quantities account for the geometry of the waveguide and should not be interpreted as conventional material density, bulk modulus, or characteristic acoustic impedance. Their use allows the corrugated waveguide to be represented by an equivalent one-dimensional model.

The wavenumber in the layer *j* is defined as

(5) $$k_{j}=\frac{\omega }{c_{j}}=\frac{2\pi f\rho_{\mathrm{eff},j}}{Z_{\mathrm{eff},j}}$$

The amplitude of the incident wave on the quasi-one-dimensional structure $A_{in}$ is related to the amplitude of the transmitted wave $A_{out}$ and the reflected wave $B_{in}$ by the relation

(6) $$\left[\begin{array}{c} A_{in}\\ B_{in} \end{array}\right]=M\left[\begin{array}{c} A_{out}\\ 0 \end{array}\right]$$

The matrix M consists of the propagation matrix $P_{j}$ in the *j* element and the transmission matrix $T_{j}$ at the boundaries of the *j* and j+1 elements

(7) $$M=\prod_{j=1}^{N}T_{j}P_{j+1}$$

The propagation matrix $P_{j}$ in element *j*, where $i=\sqrt{-1}$, is described as

(8) $$P_{j}=\left[\begin{array}{cc} e^{-ik_{j}d_{j}} & 0\\ 0 & e^{ik_{j}d_{j}} \end{array}\right]$$

The transmission matrix $T_{j}$ at the boundaries of media *j* and j+1 depends on their effective acoustic impedances $Z_{\mathrm{eff}}$ of individual elements and is defined as

(9) $$T_{j}=\frac{1}{2Z_{\mathrm{eff},j+1}}\left[\begin{array}{cc} Z_{\mathrm{eff},j+1}+Z_{\mathrm{eff},j} & Z_{\mathrm{eff},j+1}-Z_{\mathrm{eff},j}\\ Z_{\mathrm{eff},j+1}-Z_{\mathrm{eff},j} & Z_{\mathrm{eff},j+1}+Z_{\mathrm{eff},j} \end{array}\right]$$

The transmission coefficient can be determined by

(10) $$T=\frac{A_{out}}{A_{in}}=\frac{1}{M_{11}}$$

The reflection coefficient is determined as

(11) $$R=\frac{B_{in}}{A_{in}}=\frac{M_{21}}{M_{11}}$$

### 2.2. Finite-Difference Time-Domain

The propagation of a mechanical wave in a medium with effective material parameters, such as density $\rho_{\mathrm{eff}}$ and acoustic impedance $Z_{\mathrm{eff}}$ [24], is defined by the relation of the scalar pressure field *p* spread over the Cartesian space x=(x,y,z) in time *t*, and the vector field of effective instantaneous velocities v of the medium elements through a system of differential equations

(12) $$\frac{\partial p_{x,t}}{\partial t}=-\frac{Z_{\mathrm{eff}}^{2}}{\rho_{\mathrm{eff}}}\nabla \cdot v_{x,t},$$

(13) $$\frac{\partial v_{x,t}}{\partial t}=-\frac{1}{\rho_{\mathrm{eff}}}\nabla p_{x,t},$$

which for the quasi-one-dimensional case take the form

(14) $$\frac{\partial p}{\partial t}=-\frac{Z_{\mathrm{eff}}^{2}}{\rho_{\mathrm{eff}}}\frac{\partial v}{\partial x},$$

(15) $$\frac{\partial v}{\partial t}=-\frac{1}{\rho_{\mathrm{eff}}}\frac{\partial p}{\partial x},$$

In the FDTD algorithm, the above equations are discretized both spatially, with a step of Δx, and temporally, with a step of Δt, and they take the form

(16) $${\left.p\right|}_{x}^{n+\frac{1}{2}}={\left.p\right|}_{x}^{n-\frac{1}{2}}-\frac{{\left({\left.Z_{\mathrm{eff}}\right|}_{x}^{n+\frac{1}{2}}\right)}^{2}}{{\left.\rho_{\mathrm{eff}}\right|}_{x}^{n+\frac{1}{2}}}\frac{\Delta t}{\Delta x}\left({\left.v\right|}_{x+\frac{1}{2}}^{n}-{\left.v\right|}_{x-\frac{1}{2}}^{n}\right),$$

(17) $${\left.v\right|}_{x+\frac{1}{2}}^{n+1}={\left.v\right|}_{x+\frac{1}{2}}^{n}-\frac{1}{{\left.\rho_{\mathrm{eff}}\right|}_{x+\frac{1}{2}}^{n+1}}\frac{\Delta t}{\Delta x}\left({\left.p\right|}_{x}^{n+\frac{1}{2}}-{\left.p\right|}_{x-1}^{n+\frac{1}{2}}\right).$$

The spatial and temporal discretization steps were set to Δx=444μm and Δt=444ns, respectively. The Courant number is defined as

(18) $$C=\frac{c_{\max}\Delta t}{\Delta x},$$

where $c_{\max}$ is the maximum wave propagation velocity in the computational domain. For $c_{\max}=343\;m/s$, the selected discretization gives C≈0.343. The numerical stability condition C≤1 is therefore satisfied.

To reduce nonphysical reflections from the computational boundaries, absorbing layers are implemented using position-dependent multiplicative coefficients. For the *i*-th layer, the normalized spatial coordinate is defined as

(19) $$\xi_{i}=\frac{N_{\mathrm{PML}}-i}{N_{\mathrm{PML}}},$$

and the attenuation profile is calculated as

(20) $$\alpha_{i}=0.33\xi_{i}^{3}.$$

The corresponding coefficients are

(21) $$a_{i}=\frac{1}{1+\alpha_{i}},\;b_{i}=\frac{1-\alpha_{i}}{1+\alpha_{i}}.$$

The same procedure is applied at half-grid positions using

(22) $$\xi_{i+\frac{1}{2}}=\frac{N_{\mathrm{PML}}-i-0.5}{N_{\mathrm{PML}}}.$$

The resulting coefficients are applied to the pressure and particle-velocity updates, respectively, providing attenuation of outgoing waves near the computational boundaries. The implementation uses 20 absorbing layers at each boundary.

The FDTD simulations were carried out using the spatial and temporal discretization parameters specified above. Due to computational resource limitations, a systematic convergence analysis involving the refinement of the spatial and temporal grids, as well as the extension of the simulation duration, was not performed. Therefore, the influence of these parameters on the calculated transmission characteristics and Floquet sideband amplitudes was not quantitatively determined. The obtained numerical results should be treated as predictions for the adopted discretization parameters, while further convergence analysis is required to determine the numerical robustness of the results.

The acoustic source is introduced as a soft pressure source by adding a sinusoidal signal at the selected grid location. The source frequency used in the static-defect simulations is $f_{s}=2\;\mathrm{kHz}$. For dynamic-defect simulations, the effective cross-sectional area is prescribed as

(23) $$S\left(t\right)=h\left[L+A_{p}\sin\left(2\pi f_{p}t\right)\right].$$

At each time step, the corresponding effective density and acoustic impedance are updated. This method makes it possible to represent the temporal modulation of the effective acoustic parameters, while the full moving-boundary condition and the mechanical energy exchange associated with piston motion are not explicitly included in the model.

### 2.3. Sample Preparation

**Figure 1:**
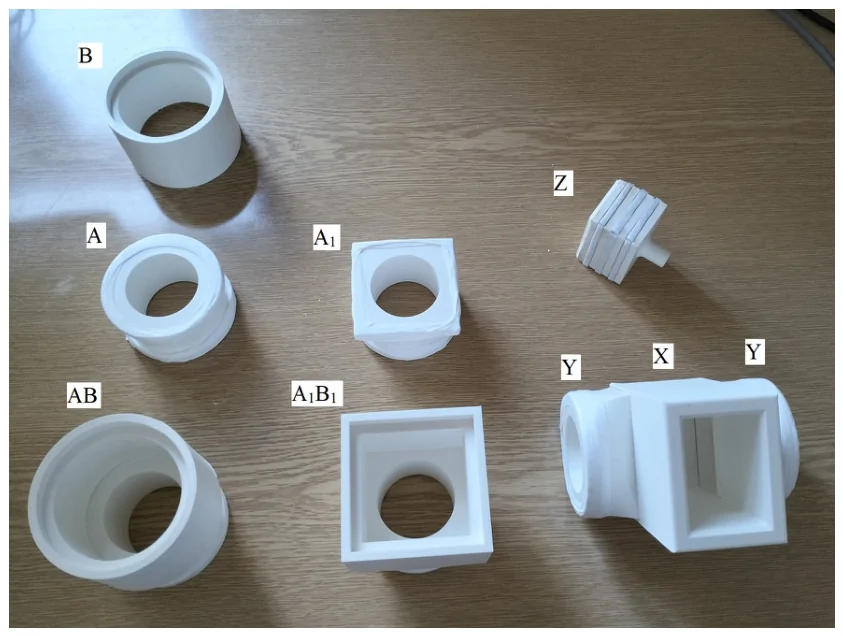
Elements of the tested phononic structure made using 3D printing technology.

In this work, a phononic crystal composed periodically of tubes with different cross-sections was analyzed. Elements of the tested phononic structure are shown in Figure 1. Elements with larger diameters were designated as B, and those with smaller diameters as A. The $A{\left(\mathrm{BA}\right)}_{7}$ structure was analyzed, where the subscript indicates the number of times the structure repeats. Then, various defects were introduced within the structure, creating ${\left(\mathrm{AB}\right)}_{3}\mathrm{YXY}{\left(\mathrm{BA}\right)}_{3}$. First, the cross-sectional shape was changed from a circle to a square instead of the elements marked as A and B, the elements $A_{1}$ and $B_{1}$ were used to create the structure ${\left(\mathrm{AB}\right)}_{3}A_{1}B_{1}A_{1}{\left(\mathrm{BA}\right)}_{3}$, with the same surface area.

**Figure 2:**
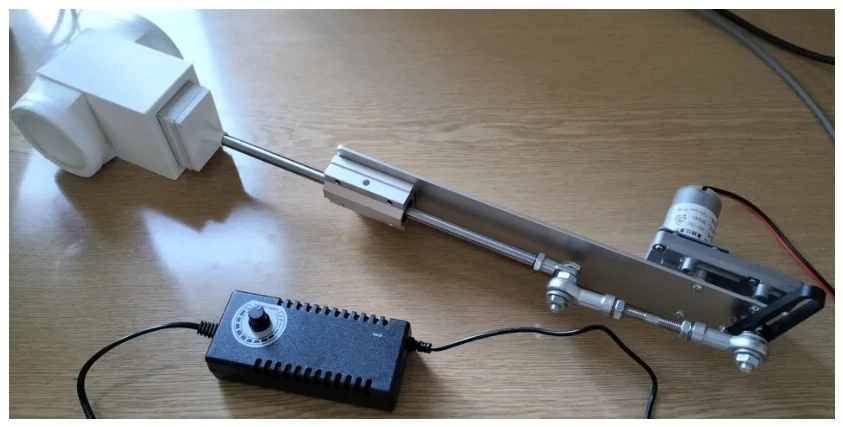
Defect YXY with dynamically varying cross–sectional area of X elements.

Finally, a defect YXY was introduced, as shown in Figure 2, creating a structure ${\left(\mathrm{AB}\right)}_{3}\mathrm{YXY}{\left(\mathrm{BA}\right)}_{3}$, which allowed for a static or dynamic change in the cross-sectional area S(L)=h·L (where h=0.06m) by moving the piston Z of the element X.

The values of the effective material parameters and the geometry of meta–atoms are summarized in Table 1.

The cross-sectional area *S* and element length *d* are geometric parameters expressed in $m^{2}$ and m, respectively. The effective density and impedance are defined as $\rho_{\mathrm{eff}}=S/c_{0}^{2}$ and $Z_{\mathrm{eff}}=S/c_{0}$, with units of $s^{2}$ and ms, respectively. These geometry-dependent quantities are used in the equivalent one-dimensional waveguide model and should not be interpreted as the conventional mass density in $\mathrm{kg}/m^{3}$ or characteristic acoustic impedance in Pas/m. The columns labeled in,out refer to the input and output sections of the modeled structure.

**Figure 3:**
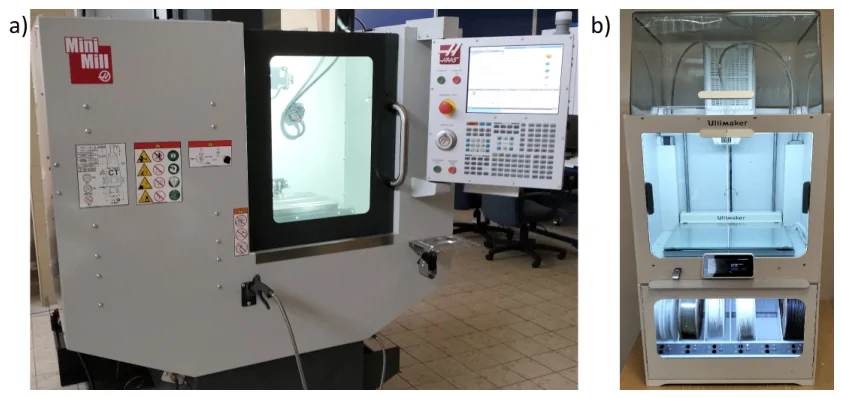
To produce the samples, (**a**) a CNC milling machine and (**b**) a 3D printer were used.

The base samples (Figure 1 and Figure 2) were made of white PLA (polylactide) material on an Ultimaker S5 Pro Bundle 3D printer (Figure 3b) and finishing of the dynamic elements in the structure was done using a Haas Mini Mill (Figure 3a).

The samples were made from Ultimaker’s white PLA filament with a density of $\rho =1.24\;g/{\mathrm{cm}}^{3}$. The filament diameter was 2.85mm, and the print nozzle diameter was 0.25mm. The printing temperature was 210 °C, and the bed temperature was 60 °C. The thickness of the layer was 0.1mm. The piston seals are made of felt with a thickness of 2mm and elements width of 5mm. The elements of the phononic structure were sealed with Teflon tape.

### 2.4. Acoustic Property Measurements

**Figure 4:**
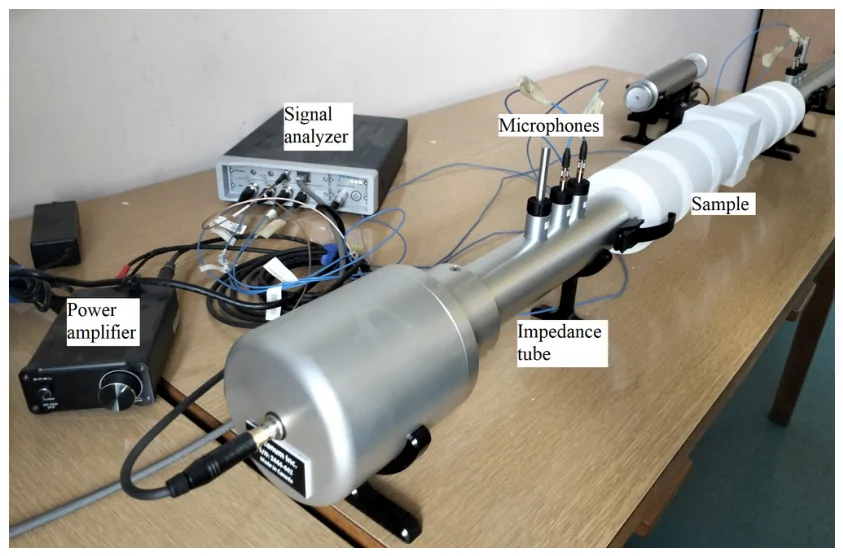
Four–microphone impedance tube with mounted ${\left(\mathrm{AB}\right)}_{3}A_{1}B_{1}A_{1}{\left(\mathrm{BA}\right)}_{3}$ structure.

The transmission of the tested structures were determined using the ASTM E2611 standard in a four-microphone impedance tube. Figure 4 shows the measurement system with the ${\left(\mathrm{AB}\right)}_{3}A_{1}B_{1}A_{1}{\left(\mathrm{BA}\right)}_{3}$ structure mounted.

The acoustic transmission of the tested structures was measured using a four-microphone impedance tube with an internal diameter of 34.9 mm. The phononic structure was installed in the main acoustic propagation path and had its own internal propagation channel. The connection between the impedance tube and the test structure was provided by custom-designed adapters manufactured using 3D printing technology. These adapters enabled the transition between the cross-section of the impedance tube and the internal channel geometry of the phononic structure.

The cross-sectional areas reported in Table 1 refer to the internal acoustic channels of the individual meta-atoms and not to the cross-sectional area of the impedance tube. Consequently, these areas may exceed the cross-sectional area of the measurement tube. The impedance tube served as part of the measurement system, whereas the acoustic wave propagated through the internal channel of the assembled phononic structure. The geometric parameters listed in Table 1 were used to define the geometry-dependent effective parameters of the equivalent one-dimensional waveguide model.

In order to reduce acoustic leakage, Teflon tape was applied at the interfaces between adjacent meta-atoms and at the connections between the assembled structure and the measurement adapters. The moving piston was additionally equipped with felt seals to limit air leakage while allowing its prescribed motion. These measures were used to improve the sealing of the assembled structure and to maintain the intended acoustic wave propagation path during the measurements.

The measurements were performed using an impedance tube and Transmission Loss and Sound Intensity Testing measurement modules from Siemens Industry Software, which includes the following:

1. Impedance Tube, model TUBE-X 34.9 mm diameter (Manufacturer: Mecanum, part number 2444-442).
2. SMSL SA36A PRO power amplifier.
3. Microphones, model 378A14 (part numbers 162991, 162992, 162993, 162994), from PCB Piezotronics Inc.
4. Simcenter SCADAS SCM2E01 4-channel analyzer (part number 21233908), with an SCM-V4E measurement module (part number 2023044028).
5. Simcenter Testlab 2406 measurement platform.

The experimental spectra were determined using a Hanning window, RMS scaling and energy averaging based on 121 realizations. These realizations were used to perform spectral averaging and did not represent independent repetitions of the entire measurement procedure. Therefore, the variability between independent measurements could not be directly determined.

The uncertainties associated with the piston’s equilibrium position *L* and the amplitude of its motion $A_{p}$ were estimated at 0.5 mm for each parameter. These uncertainties affect the effective cross-sectional area of the defect, described by the Equation (23), where h=0.06 m.

## 3. Results

In this part of the work, transmission spectra for static structures were first determined using the TMM algorithm and compared with experimentally obtained spectra. The influence of defect geometry within the structure on the transmission spectrum was examined. Next, the influence of static defects on mechanical wave propagation within the structure was investigated using the FDTD algorithm. The final step was numerical (FDTD) and experimental analysis of the structure with the dynamic defect.

### 3.1. Static Defect—TMM and Experiment

**Figure 5:**
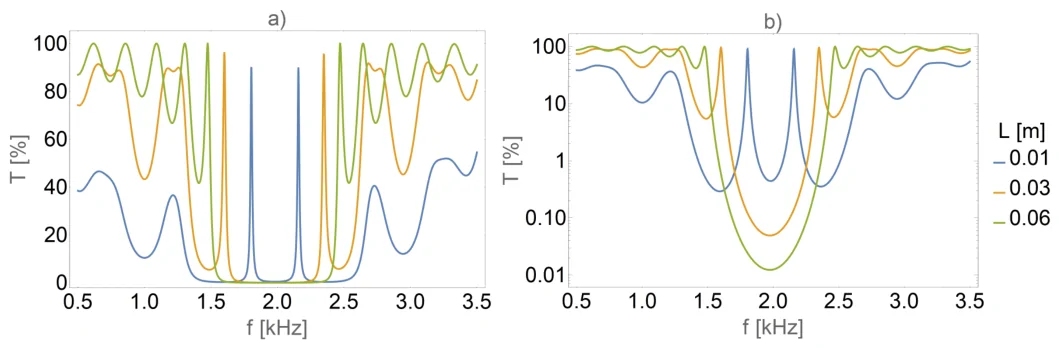
Transmission of the quasi-static structure on (**a**) linear and (**b**) logarithmic scales for different values of L oin the defect.

The first element of the analysis was to determine the transmission spectra for the designed structure using the TMM algorithm. To better illustrate the effect of changes in the cross-sectional area S(L) on the transmission spectrum, graphs are presented both on a linear scale (Figure 5a) and on a logarithmic scale (Figure 5b).

**Figure 6:**
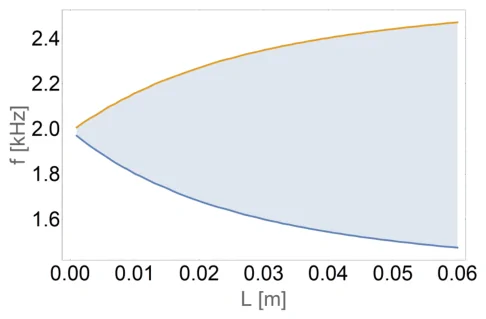
Positions of the transmission peaks surrounding the fundamental band gap region as a function of the defect parameter L.

The TMM algorithm assumes effective values of acoustic impedance and density, which include the surface area but does not take into account the shape of this field. For parameter L=0.06m, the structure $A{\left(\mathrm{BA}\right)}_{7}$ does not contain a defect, and a clear fundamental band gap can be observed around frequency 2kHz. Decreasing the value of the L parameter introduces a defect X into the structure ${\left(\mathrm{AB}\right)}_{3}\mathrm{YXY}{\left(\mathrm{BA}\right)}_{3}$ and causes a decrease in the width of the fundamental band gap, as shown in Figure 6.

In this study, the influence of the defect parameter L on the fundamental band-gap region is assessed from the calculated and measured transmission spectra. The main low-transmission frequency interval is identified from the overall shape of the transmission spectra, whereas the positions of the neighboring transmission peaks and minima are analyzed separately. The plotted peak positions characterize the changes in the transmission response induced by the defect and should not be interpreted as direct measurements of the band-gap boundaries. Localized transmission resonances may occur in the vicinity of the fundamental band gap without necessarily indicating a shift in its boundaries.

**Figure 7:**
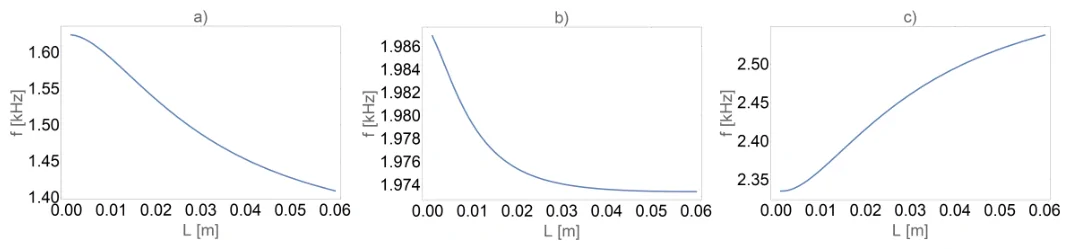
Minimum shifts in (**a**) the fundamental band gap and (**b**); (**c**) the side bands depending on the parameter L.

The formation of high transmission peaks and band gaps on both sides of the fundamental band gap was observed. With the change in the L parameter, there was a slight shift in the fundamental band gap minimum and a significant shift in the sideband minima (Figure 7).

**Figure 8:**
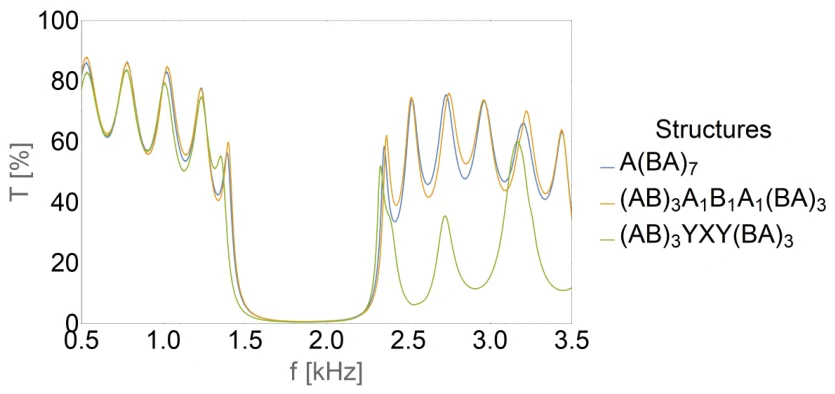
Influence of the shape of the analyzed defects on the transmission spectrum.

The next step was to experimentally investigate the effect of defect shape in the same cross-sectional area upon the occurrence of the band gap. Figure 8 shows the measured transmission spectra. As can be seen, changing the cross-section from circular (element B) to square (element $B_{1}$), when these elements were aligned with the structure, did not significantly affect transmission. The minor changes were caused by modifications to the modal impedance and local wave propagation conditions. The mode mismatch at the defect boundaries resulted in changes in the phase and amplitude of the reflection and transmission coefficients. This led to a shift in the defect resonance frequencies and affected the transmission characteristics of the phononic crystal. These effects had a much greater impact on transmission for the defect adapted to dynamic changes due to significant changes in geometry—switching from circular to rectangular cross-sections (replacing element A with element Y and element B with element X) despite maintaining the same cross-sectional area of the elements on both sides of the defect. Despite the reduction in transmission at higher frequencies, the band gap occurred in the same frequency range.

**Figure 9:**
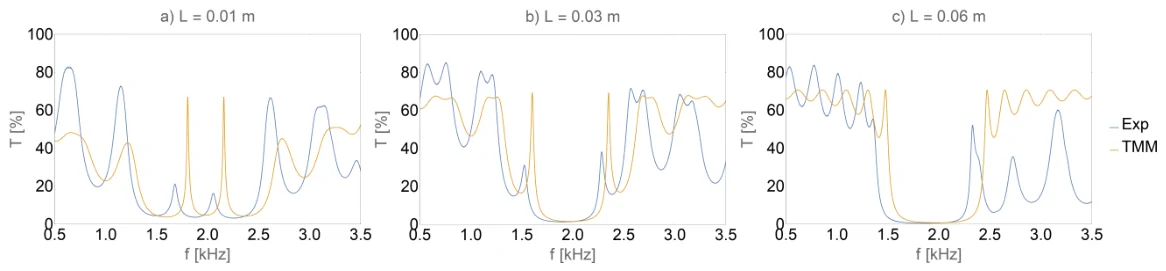
Comparison of transmission spectra obtained analytically (TMM) and experimentally (Exp) for selected dynamic defect positions.

Figure 9 shows a comparison of the transmission spectra obtained using the TMM (Transfer Matrix Method) algorithm and those obtained experimentally for different values of the parameter L. This comparison demonstrated qualitative agreement in the primary band gap region, although differences in the positions and amplitudes of individual transmission extrema were observed, particularly in the frequency ranges adjacent to the main band gap. The conducted analysis showed that the idealized TMM model correctly reproduced the main frequency characteristics of the structure, but did not fully reproduce its detailed transmission response.

Several factors may contribute to these differences, such as acoustic dissipation, manufacturing deviations, or imperfect sealing between the individual structural components. Furthermore, the simplified one-dimensional model does not explicitly account for all the geometric and modal effects that can influence wave propagation in a real-world system. However, the available measurement data do not allow for a quantitative determination of the specific contribution of each mechanism to the observed discrepancies. Consequently, this comparison is interpreted primarily in the context of the main features of the band gap, rather than in terms of precise alignment of resonant frequencies or transmission amplitudes. A more detailed quantitative assessment would require a systematic identification of corresponding spectral extrema, as well as additional measurements to characterize experimental variability and geometric uncertainties.

**Figure 10:**
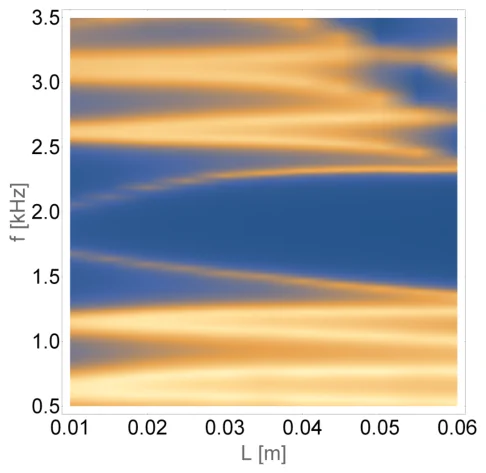
The experimental transmission spectra as a function of the L parameter.

Figure 10 shows the transmission spectra as a function of the L parameter. As can be seen, the fundamental band gap narrowed with the reduction of the defect cross-sectional area, and the formation of the side bands predicted in Figure 5 and Figure 6.

### 3.2. Static Defect—FDTD

The TMM algorithm allows for the study of transmission spectra behind a phononic structure. To investigate wave propagation within the analyzed static structures, the FDTD algorithm was used. A soft source at location *x* was defined by

(24) $${\left.p\right|}_{x}^{n+1}={\left.p\right|}_{x}^{n}+\sin\left(2\pi f_{s}n\Delta t\right),$$

and a frequency $f_{s}$ within the forbidden region of 2kHz was used. To prevent the wave from reflecting into the simulation region, 20 PML layers were applied at the simulation boundaries. Space and time discretizations were performed, for which the corresponding steps were Δx=444 µm and Δt=444ns. Simulations were performed for $n=5\cdot 10^{5}$ time steps, resulting in a propagation time of 222ms. The analysis was performed for the analogous values of the *L* parameter as in the TMM method.

**Figure 11:**
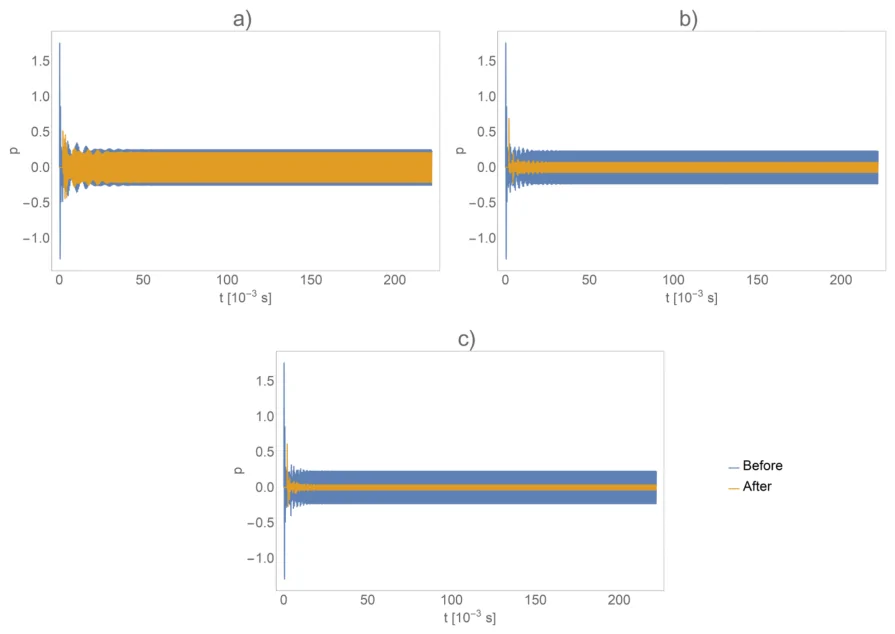
Time series of pressure before and after the structure for parameter L equal to (**a**) 0.01m, (**b**) 0.03m and (**c**) 0.06m, respectively.

**Figure 12:**
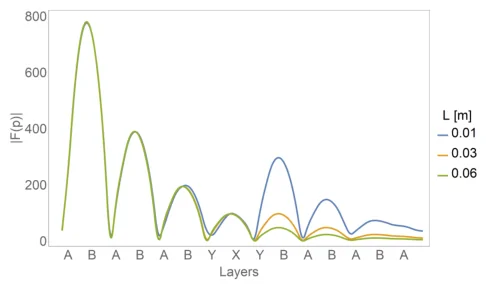
Distribution of the pressure amplitude of a mechanical wave with frequency 2kHz inside the phononic structure with static defect.

Figure 11 presents time series before and after the structure for parameter *L* equal to (a) 0.01m, (b) 0.03m, and (c) 0.06m, respectively. Stabilization of the propagating mechanical wave occurred after $75\cdot 10^{3}$ time steps. When the structure reflected a phononic crystal without a defect (Figure 11c), the greatest reduction in amplitude occurred, which can also be observed in Figure 12. Introducing a structure defect in the form of a reduced cross–sectional area of element X increased the wave transmission by creating local resonance regions behind the defect, visible in Figure 12.

**Figure 13:**
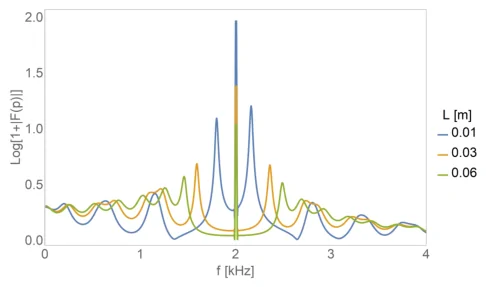
Spectra of the wave propagating behind the structure with static defect on a logarithmic scale.

Figure 13 presents the spectra of the wave propagating behind the structure on a logarithmic scale. As can be seen, for the finite phononic structure, despite operating within the forbidden region, the spectrum contains a component corresponding to the wave source frequency (2kHz). Interestingly, transmission peaks at the forbidden region boundaries can also be observed in the spectrum, corresponding to the peaks obtained from the TMM simulation in Figure 5 and those observed experimentally (Figure 9).

**Figure 14:**
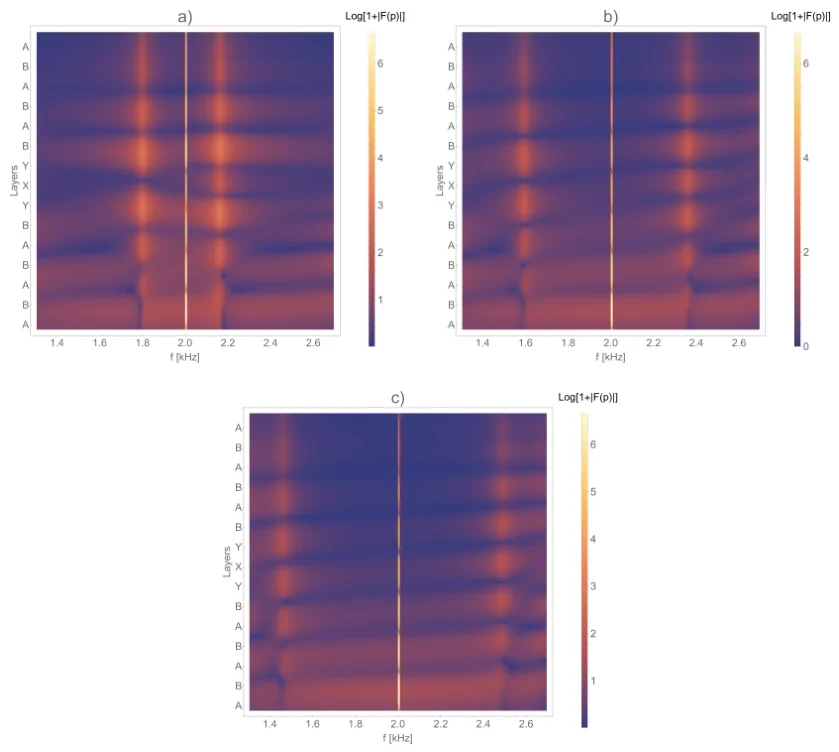
Distribution of spectra inside a phononic crystal with a static defect for parameter L equal to (**a**) 0.01m, (**b**) 0.03m and (**c**) 0.06m, respectively.

Figure 14 shows the spectral distributions obtained from the FDTD algorithm inside a phononic crystal with a static defect. Peak positions at the gap boundaries and a high-intensity peak associated with the wave source were observed, the amplitude of which decreased inside the structure, as shown in Figure 12.

### 3.3. Dynamic Defect—FDTD and Experiment

The next step was to introduce a dynamic defect and analyze the effect of its vibrations on the propagation of the mechanical wave.

By introducing an element X with effective time-varying parameters, the Equations (16) and (17), for this defect, take the form

(25) $${\left.p\right|}_{x}^{n+\frac{1}{2}}={\left.p\right|}_{x}^{n-\frac{1}{2}}-\frac{h\left(L+A_{p}\;\sin\left(2\pi f_{p}n\Delta t\right)\right)\Delta t}{\Delta x}\left({\left.v\right|}_{x+\frac{1}{2}}^{n}-{\left.v\right|}_{x-\frac{1}{2}}^{n}\right),$$

(26) $${\left.v\right|}_{x+\frac{1}{2}}^{n+1}={\left.v\right|}_{x+\frac{1}{2}}^{n}-\frac{c_{0}^{2}\Delta t}{h\left(L+A_{p}\;\sin\left(2\pi f_{p}\left(n+\frac{1}{2}\right)\Delta t\right)\right)\Delta x}\left({\left.p\right|}_{x}^{n+\frac{1}{2}}-{\left.p\right|}_{x-1}^{n+\frac{1}{2}}\right),$$

where L defines the equilibrium point of the piston Z, $A_{p}$ is the amplitude of its motion, and $f_{p}$ is the modulation frequency.

The update equations are obtained by substituting the time-dependent effective parameters Equations (3) and (4) into the discretized acoustic equations. Consequently, the coefficients satisfy $Z_{\mathrm{eff}}^{2}\left(t\right)/\rho_{\mathrm{eff}}\left(t\right)=S\left(t\right)$ and $1/\rho_{\mathrm{eff}}\left(t\right)=c_{0}^{2}/S\left(t\right)$, which gives the time-dependent terms appearing in Equations (25) and (26). The pressure and particle-velocity updates use the corresponding discrete-time values of the modulated cross-sectional area, consistent with the staggered temporal discretization of the FDTD scheme.

The time-dependent perturbation was introduced by defining an effective cross-sectional area as Equation (23). The corresponding effective parameters are updated at each time step, which introduces temporal modulation into the coefficients of the discretized acoustic equations. However, the full kinematic boundary condition related to the piston motion and the mechanical work exchanged between the piston and the acoustic field are not taken into account. Therefore, the model was used to investigate frequency conversion caused by the prescribed time modulation of parameters rather than to describe the complete coupled piston–acoustic field system. The influence of the neglected effects associated with the motion of the boundaries represents a limitation of the proposed formulation.

Due to mechanical constraints of the experimental setup, the modulation frequency ranges differed between the numerical and experimental studies. FDTD simulations were conducted for modulation frequencies in the range of 5–200 Hz, while experimental measurements were performed at 0.25 Hz, 1.6 Hz, and 2.2 Hz, in addition to a static case (reference, $f_{p}=0$). The piston displacement amplitude $A_{p}$ was set to 0.01 m for the numerical simulations and 0.017 m for the experiments. In the experimental setup, the dynamic defect was driven by a direct current (DC) motor. The approximately sinusoidal motion of the piston resulted from the mechanical design of the device, rather than from a sinusoidal electrical control signal. Consequently, the actual motion in the experiment was determined by the mechanical response of the motor and its associated components, whereas the numerical model assumed an ideal, predetermined sinusoidal modulation.

The experimental results demonstrated the occurrence of frequency conversion within the analyzed modulation range. However, the obtained results do not allow for direct verification of the numerical predictions in the frequency range from 5 Hz to 200 Hz. Moreover, the comparison of the spectral characteristics requires taking into account the differences in piston displacement amplitudes used in the numerical and experimental studies. Therefore, further experimental analysis using a mechanical actuator operating in the appropriate frequency range is required to verify the effects predicted for higher modulation frequencies.

#### The Influence of Modulation Frequency on Mechanical Wave Propagation

The first analysis was performed for low values of the modulation frequency $f_{p}$, which are possible to generate in the analyzed mechanical systems. Higher frequency ranges would require a completely different design of the dynamic defect. In the analyzed cases, the value of the parameter L was 0.03m and $A_{p}$ was 0.01m.

**Figure 15:**
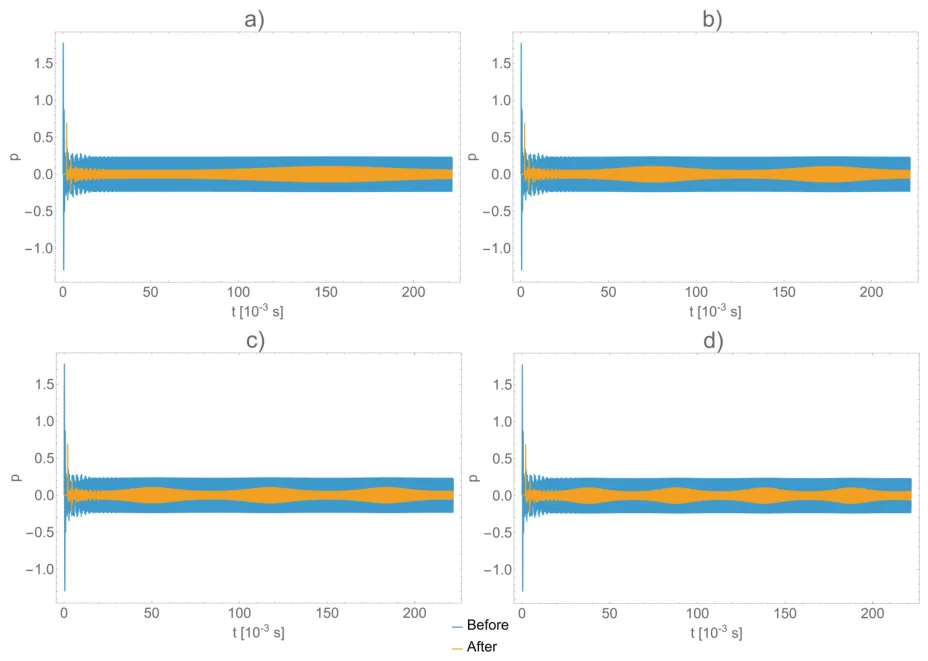
Time series of pressure before and after the structure for parameter L=0.03m and $f_{p}$ equal to (**a**) 5Hz, (**b**) 10Hz, (**c**) 15Hz and (**d**) 20Hz, respectively.

Figure 15 presents the time series of pressure behind the dynamic structure. The introduction of low values of the modulation frequency $f_{p}$ (5Hz, 10Hz, 15Hz and 20Hz) for the dynamic defect did not introduce any changes in the stabilization time of the mechanical wave propagation, but a clear amplitude modulation of the wave emerging from the structure can be observed, related to the frequency of changes in $f_{p}$.

**Figure 16:**
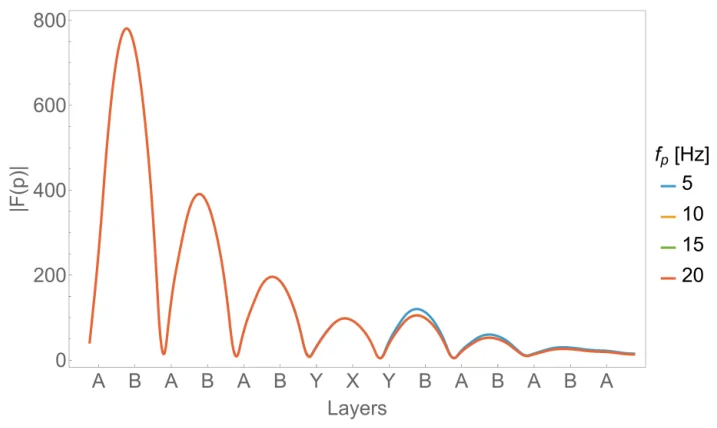
Distribution of the pressure amplitude of a acoustic wave with frequency 2kHz inside the phononic structure with dynamic defect for values of the modulation frequency $f_{p}$ equal to 5Hz, 10Hz, 15Hz and 20Hz.

**Figure 17:**
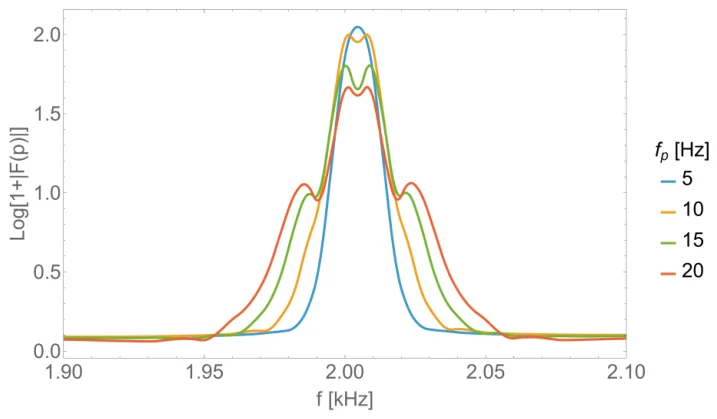
Spectra of the wave propagating behind the structure with a low-frequency dynamic defect at a logarithmic scale.

**Figure 18:**
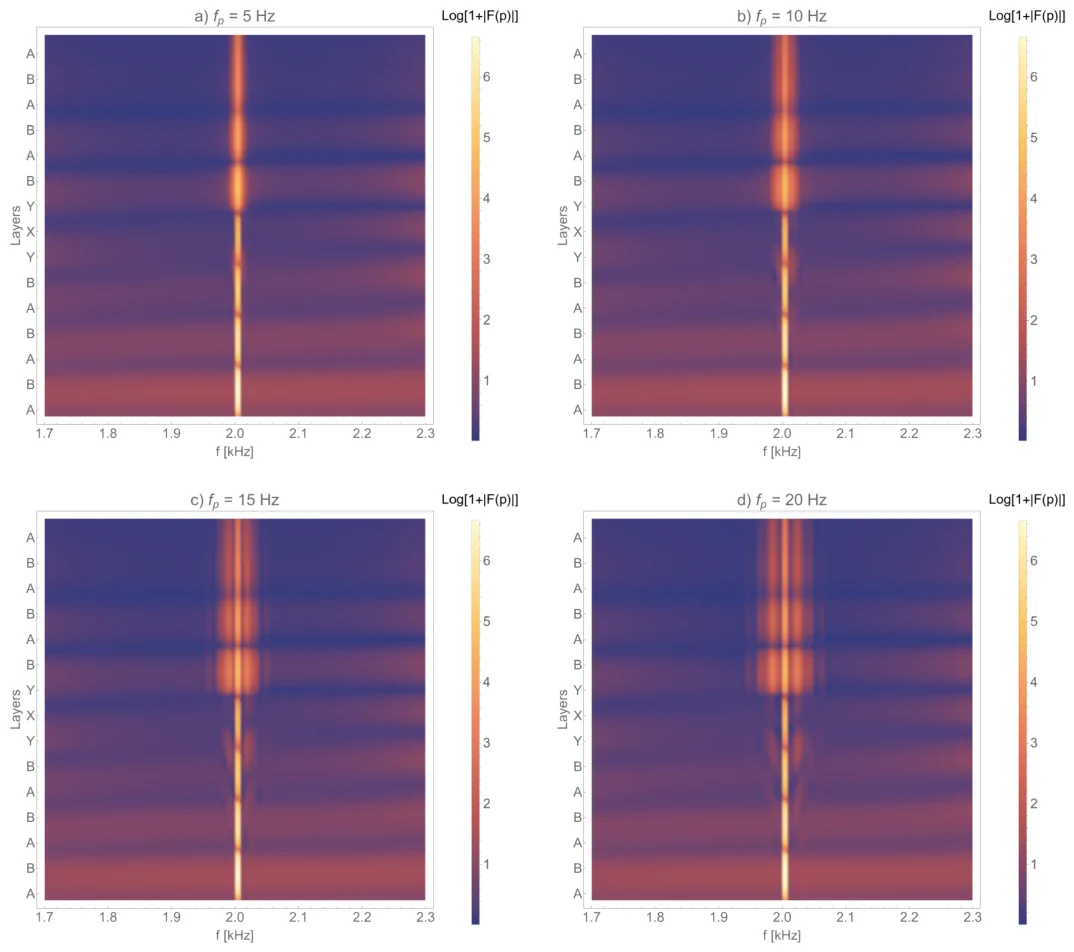
Distribution of spectra inside a phononic crystal with a low-frequency dynamic defect at a logarithmic scale.

The introduction of a dynamic defect had almost no effect on the local resonance region behind the defect shown in Figure 16. However, it decreased the peak amplitude in the spectrum of the mechanical wave propagating behind the structure (Figure 17) and additional peaks appeared related to the modulation frequency. Floquet harmonics occur at frequencies defined by Equation (1), where $f_{s}$ denotes the source frequency, $f_{p}$ is the modulation frequency, and *k* is an integer. As shown in Figure 18, Floquet harmonics appear mainly behind the dynamic defect at low values of the modulation frequency.

**Figure 19:**
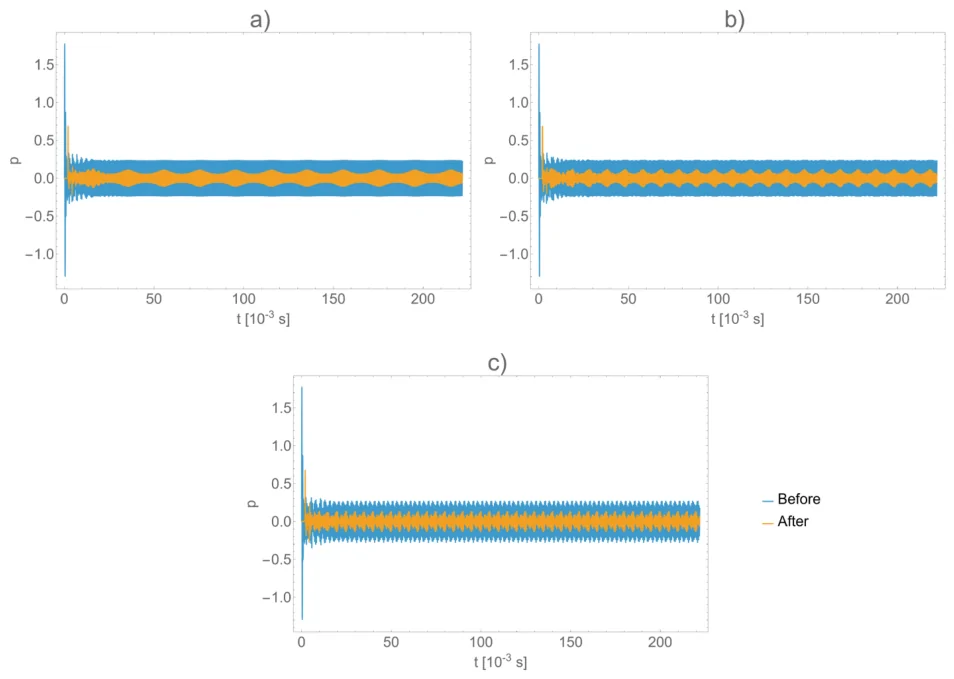
Time series of pressure before and after the structure for parameter L=0.03m and $f_{p}$ equal to (**a**) 50Hz, (**b**) 100Hz and (**c**) 200Hz, respectively.

**Figure 20:**
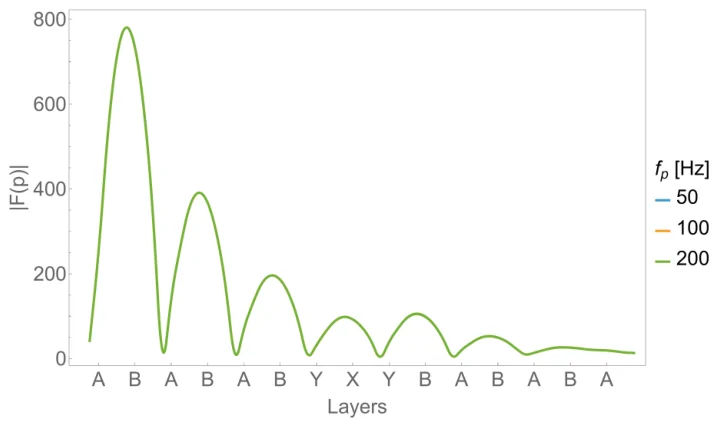
Distribution of the amplitude of a wave with frequency 2kHz inside the phononic structure with dynamic defect for values of the modulation frequency $f_{p}$ equal to 50Hz, 100Hz and 200Hz.

The last element of the analysis of the influence of modulation frequency on mechanical wave propagation in a structure with a dynamic defect was to investigate values that were physically impossible to obtain in the designed structure. The study was carried out for $f_{p}$ with values equal to 50Hz, 100Hz and 200Hz. A significant increase in the modulation frequency increased the amplitude modulations of the mechanical wave propagating behind the structure (Figure 19), and for values of $f_{p}$ equal to 100Hz and 200Hz introduced modulations of the amplitude of the wave reflected from the structure. However, this did not affect the distribution of local wave amplitude resonances at the source frequency inside the structure in any way (Figure 20).

**Figure 21:**
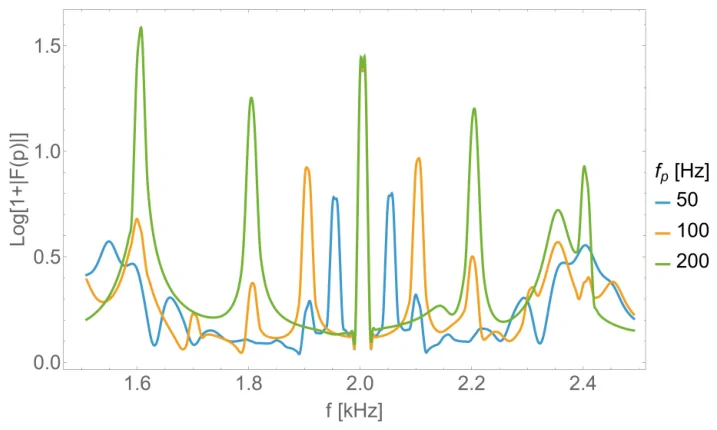
Spectra of the wave propagating behind the structure with a high-frequency dynamic defect at a logarithmic scale.

**Figure 22:**
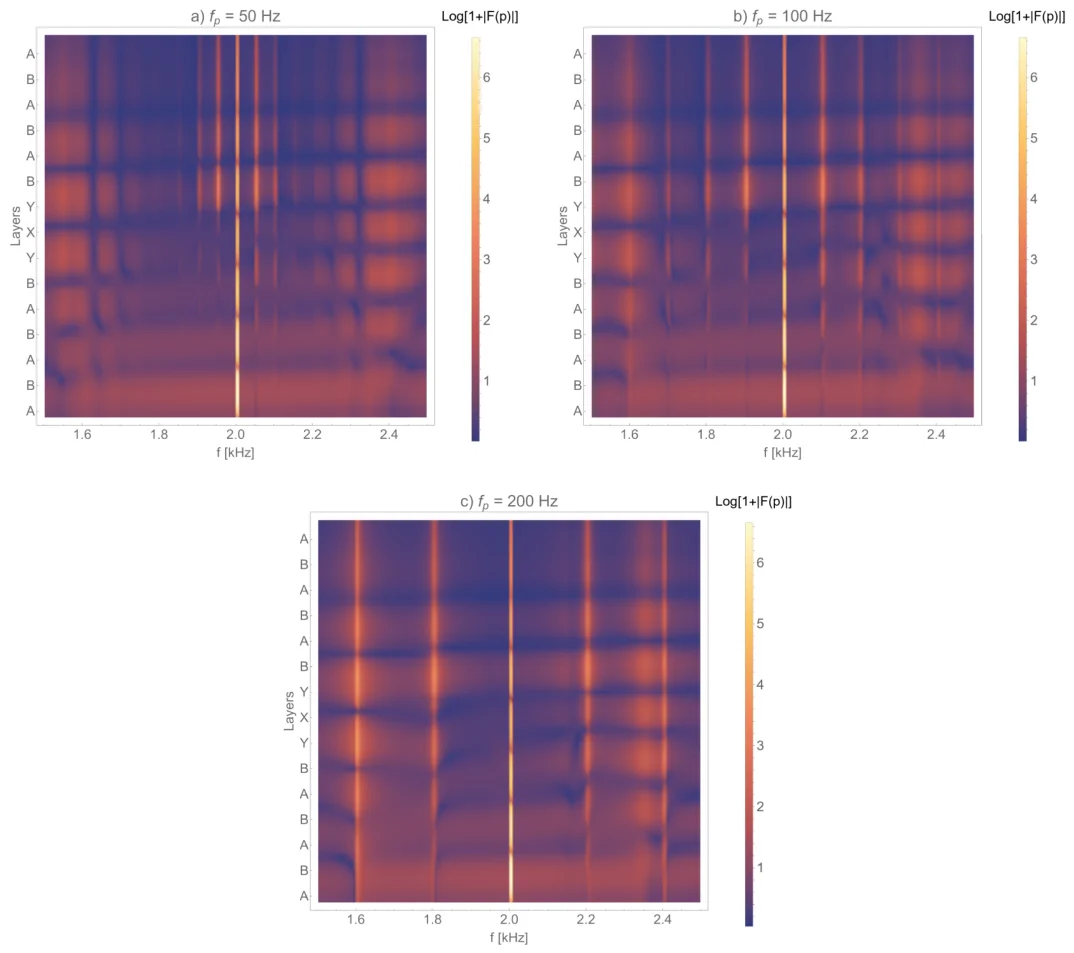
Distribution of spectra inside a phononic crystal with a high-frequency dynamic defect at a logarithmic scale.

Floquet harmonics are clearly visible in Figure 21, and for higher modulation frequency values they also appear as spectral components in front of the dynamic defect as a component of the reflected wave, which can be observed in Figure 22.

### 3.4. The Influence of the Piston Motion Amplitude on the Mechanical Wave Propagation

Then, an analysis of the effect of the piston vibration amplitude on the mechanical wave propagation was performed. The value of the parameter L was 0.03m, the modulation frequency was $f_{p}=20\;\mathrm{Hz}$ and the piston motion amplitude $A_{p}$ was 0.015m, 0.020m and 0.025m.

**Figure 23:**
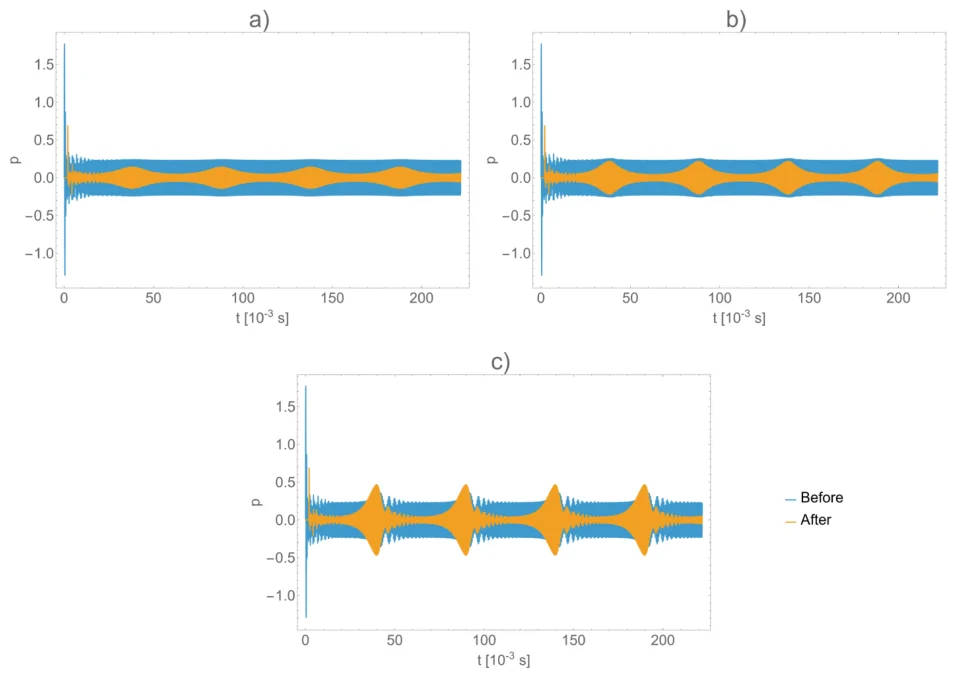
Time series of pressure in front of and behind the structure for different values of the piston motion amplitude $A_{p}$ equal to (**a**) 0.015m, (**b**) 0.020m and (**c**) 0.025m.

**Figure 24:**
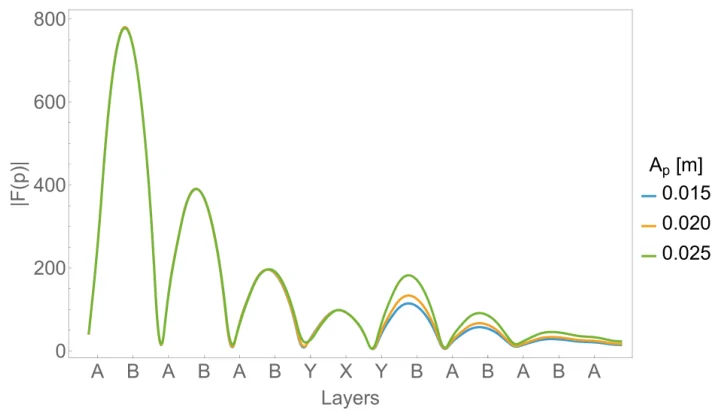
Distribution of the amplitude of a wave with frequency 2 kHz inside the phononic structure with a dynamic defect for different values of the piston motion amplitude $A_{p}$.

**Figure 25:**
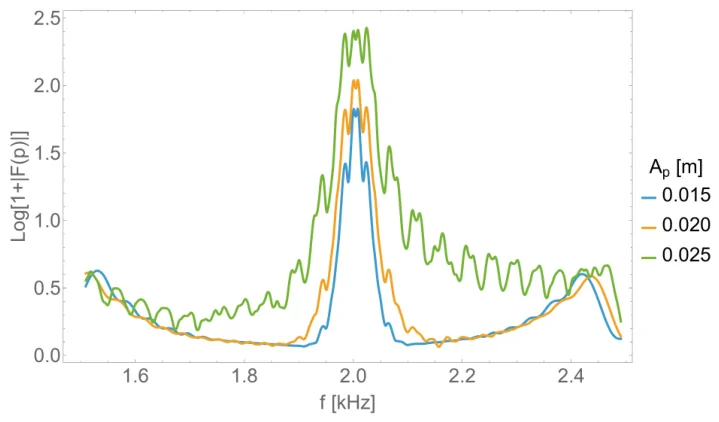
Spectra of the wave propagating behind the structure for different values of the piston motion amplitude $A_{p}$.

**Figure 26:**
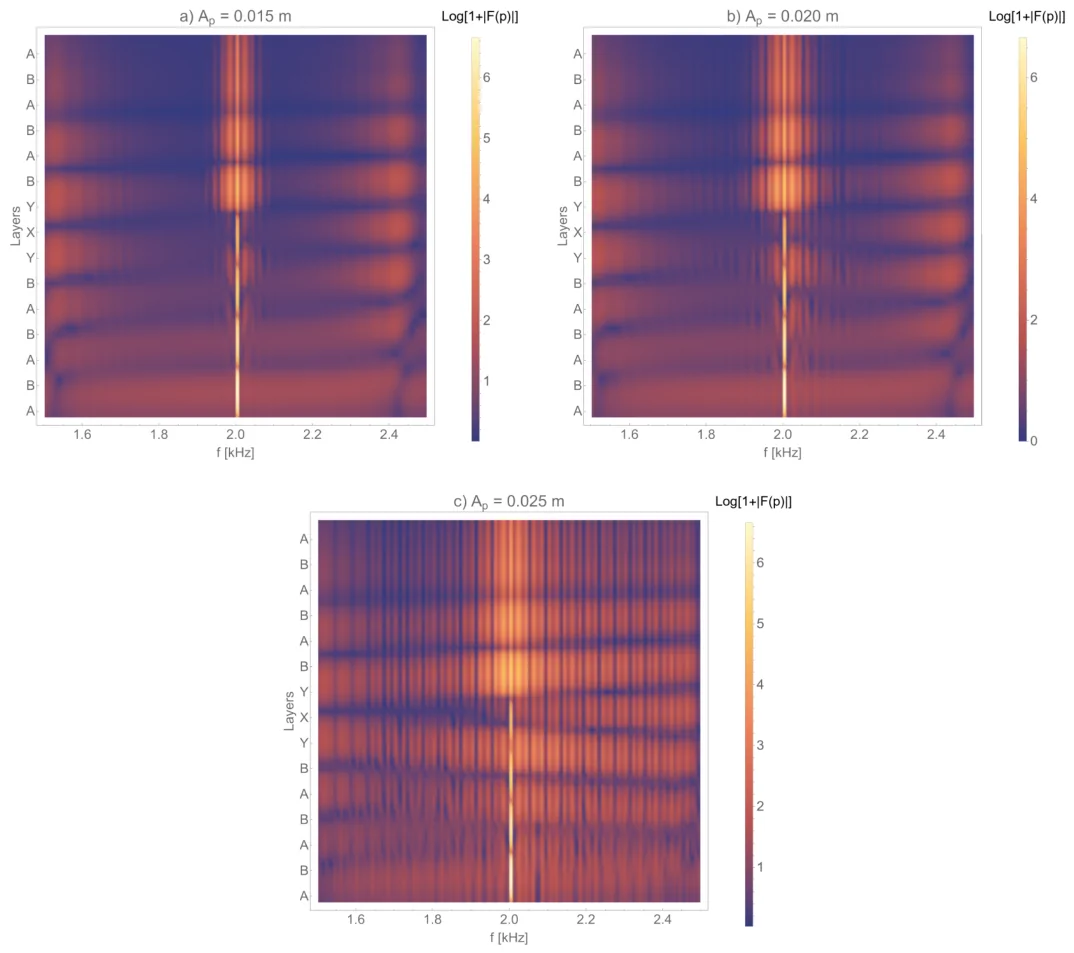
Distribution of spectra inside a phononic crystal with dynamic defect for different values of the piston motion amplitude $A_{p}$.

Increasing the amplitude of the piston motion $A_{p}$ increased the amplitude of the modulation wave transmitted by the structure, and for the value 0.025m it also significantly affected the pressure time series of the reflected wave (Figure 23). With the increase of the modulation amplitude, the amplitude of the local resonance region behind the dynamic defect increased, as shown in Figure 24. One can also notice an increase in the amplitudes of the transmitted wave spectrum, a broadening of the half-width of the peaks, and intensification in the spectrum of Floquet harmonics (Figure 25). These phenomena occur within the entire structure (Figure 26) for each amplitude increase, although they are most visible for the case of the highest amplitude jump (Figure 26c).

### 3.5. Experimental Analysis

The structure ${\left(\mathrm{AB}\right)}_{3}\mathrm{YXY}{\left(\mathrm{BA}\right)}_{3}$ was analyzed, for which L=0.03m. Due to the mechanical limitations of the dynamic defect shown in Figure 2, the tests were performed for modulation frequencies $f_{p}$ equal to 0Hz (static defect), 0.25Hz (the lowest physically possible frequency for the used motor), 1.6Hz and 2.2Hz (the highest frequency for the used motor).

The modulation frequencies used in the experiments were lower than those applied in the FDTD simulations due to the mechanical limitations of the actuator used in the experimental setup. In the numerical analysis, the frequency range of $f_{p}$ = 5–200 Hz was considered, while the experimental studies were carried out for $f_{p}=0.25$, 1.6, and 2.2 Hz, as well as for the static case ($f_{p}=0$). Therefore, the experimental results did not allow for direct quantitative verification of the numerical predictions over the entire analyzed frequency range. However, the conducted research demonstrated the occurrence of frequency conversion and its dependence on the temporal modulation of a local defect.

For an excitation frequency of $f_{s}=2000$ Hz, periodic modulation should result in the emergence of spectral components at frequencies $f_{k}=f_{s}+kf_{p}$, where *k* is an integer. The predicted first-order sidebands appear at frequencies of 1999.75 and 2000.25 Hz for $f_{p}=0.25$ Hz; 1998.4 and 2001.6 Hz for $f_{p}=1.6$ Hz; and 1997.8 and 2002.2 Hz for $f_{p}=2.2$ Hz. Higher-order sidebands should appear at distances from the source frequency that are integer multiples of the corresponding modulation frequency. The measured positions of spectral components can therefore be compared with these predicted values to assess their consistency with a Floquet-type frequency conversion mechanism.

To investigate the potential influence of mechanical noise, background measurements were conducted with the acoustic source turned off, while the dynamic defect was operated at selected modulation frequencies. These measurements serve as a reference, allowing for the identification of spectral components associated with the actuator itself and the measurement system. When interpreting additional peaks in the spectrum, both the spectra obtained during excitation and the background spectra must be considered. Spectral components appearing near the predicted Floquet frequencies—which can be distinguished from the background—provide stronger evidence for the occurrence of frequency conversion than individual spectral peaks considered in isolation.

The spectral resolution obtained in the conducted experiments was 0.0488 Hz. Although this value was lower than the spacing between adjacent Floquet harmonics for all non-zero modulation frequencies considered in the experiment, the sidebands generated at $f_{p}=0.25$ Hz were located close to each other, which could lead to their overlap in the recorded spectral response. The apparent broadening of the sidebands could also result from the influence of the spectral window, the finite data acquisition time and the measurement noise. Therefore, the experimental results were analyzed primarily in terms of the occurrence and distribution of additional spectral components, rather than as a complete quantitative verification of the sideband amplitudes obtained from the FDTD simulations.

Experimental acoustic characteristics were determined using the Simcenter Testlab 2406 system, recording sound pressure at a sampling rate of 6400 Hz. Frequency analysis was performed over the 0–3200 Hz range, with a resolution of 0.0488 Hz. The pressure spectrum was determined using a Hanning window, RMS scaling, and energy averaging of 121 realizations. The signal was measured using a pressure transducer with a sensitivity of 1.12993 mV/Pa.

**Figure 27:**
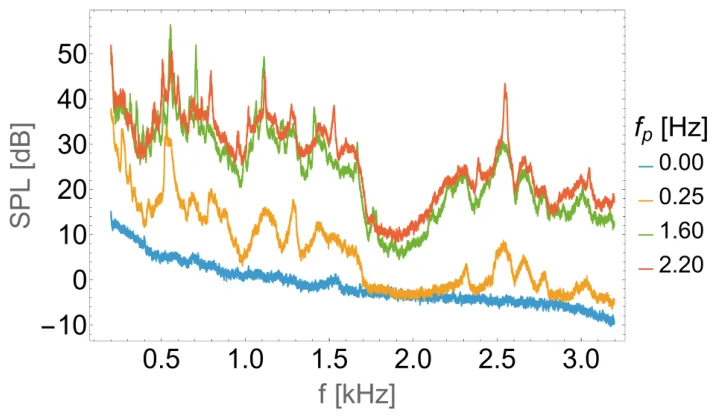
Sound pressure level for a defect operating at a given modulation frequency with the source turned off.

**Figure 28:**
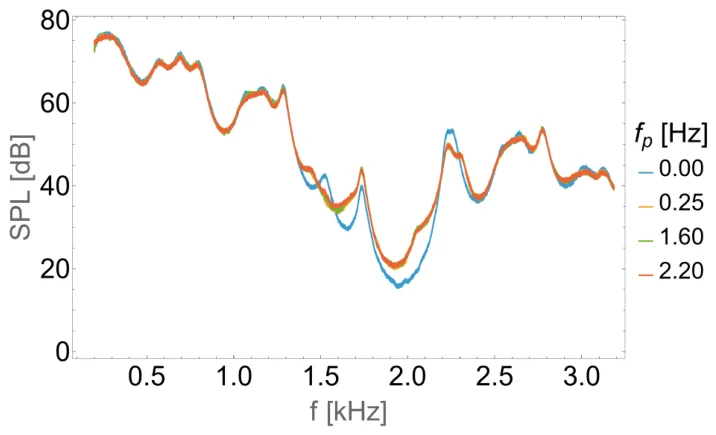
Sound pressure level for a dynamic defect operating at a given modulation frequency with white noise generated.

**Figure 29:**
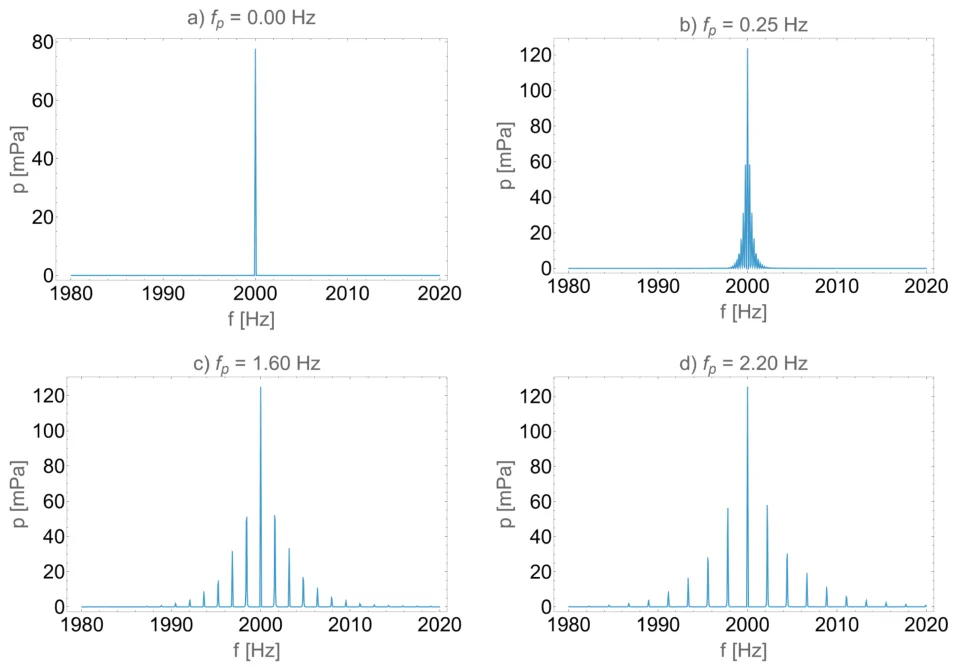
Formation of Floquet harmonics in the spectrum depending on the modulation frequency.

**Figure 30:**
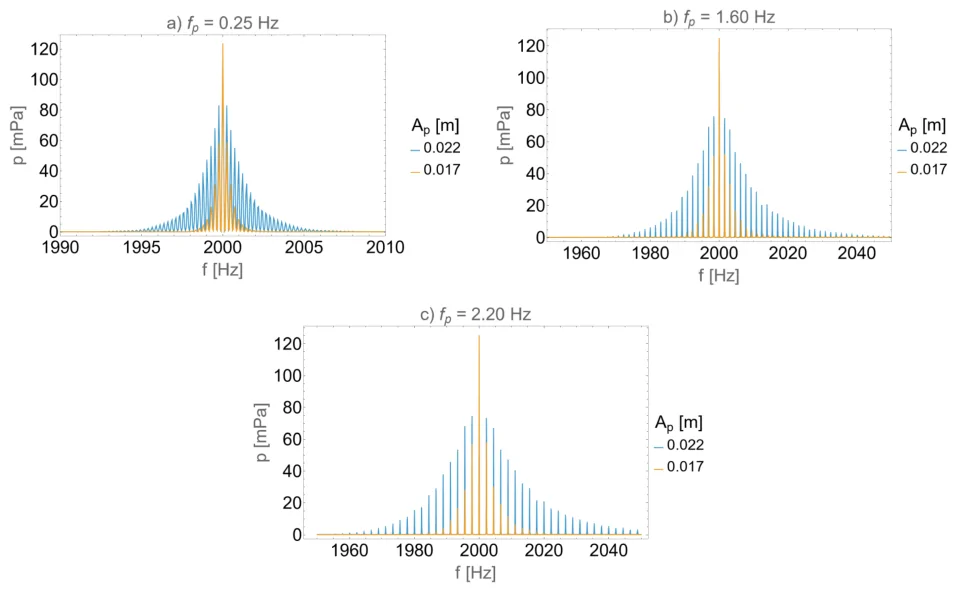
Influence of the piston operating amplitude on the spectrum of the transmitted mechanical wave.

Using the Sound Intensity Testing measurement module of the Simcenter Testlab 2406 environment, the sound pressure level was measured at the third microphone of the impedance tube (behind the structure) of the working defect with the source turned off (Figure 27), with generated white noise (Figure 28) and a generated acoustic wave with frequency $f_{s}=2\;\mathrm{kHz}$ (Figure 29 and Figure 30).

The values in the graphs in Figure 27 and Figure 28 are presented after smoothing with the exponential weighted average filter for better readability, while the graphs in Figure 29 and Figure 30 were not smoothed.

Figure 27 shows the acoustic background generated by the tested dynamic defect depending on the piston operating frequency. With increasing engine speed, the background values increased, although significantly lower acoustic pressure levels were observed within the analyzed band gap range.

The tested band gap also appeared in the spectrum of the transmitted wave when the source generated white noise (Figure 28). It should be noted that the sound pressure values for the operating system inside the band gap were higher than for the static system, which was undoubtedly influenced by the system noise, but did not depend, in the tested range, on the piston operating frequency.

To quantify the experimentally observed frequency conversion, the amplitudes of the first-order Floquet sidebands were evaluated relative to the transmitted component at the source frequency, $f_{s}=2000\;\mathrm{Hz}$. The normalized sideband amplitude was defined as

(27) $$R_{\pm 1}=\frac{\left|p(f_{s}\pm f_{p})\right|}{\left|p(f_{s})\right|}\times 100\%,$$

where p(f) denotes the measured acoustic pressure amplitude at frequency *f*. The sideband amplitudes were identified from the spectral peaks nearest to the expected frequencies $f_{s}-f_{p}$ and $f_{s}+f_{p}$, taking into account the finite frequency resolution of the measurement system.

For $f_{p}=0.25\;\mathrm{Hz}$, the source-frequency amplitude was 122.72mPa, while the amplitudes of the lower and upper first-order sidebands were 57.20 and 57.40mPa, respectively. The corresponding mean normalized amplitude was 46.69%. For $f_{p}=1.6\;\mathrm{Hz}$, the source-frequency amplitude was 124.00mPa, and the lower and upper sideband amplitudes were 50.29 and 51.19mPa, respectively, giving a mean normalized amplitude of 40.92%. For $f_{p}=2.2\;\mathrm{Hz}$, the corresponding sideband amplitudes were 55.46 and 57.05mPa, resulting in a mean normalized amplitude of 45.21%.

An analysis of the measured spectral components in Table 2 revealed that the amplitudes of the first-order sidebands reached approximately 41–47% of the source frequency amplitude within the investigated frequency range. It was found that the dependence of the normalized sideband amplitude on the modulation frequency is non-monotonic, meaning that a higher modulation frequency does not necessarily result in stronger first-order sidebands. At the same time, the modulation frequency determined the spacing between successive Floquet components and influenced the amplitude values obtained from the measurements.

The static configuration ($f_{p}=0$) exhibited a dominant spectral component at the source frequency. The neighboring spectral bins were not interpreted as Floquet sidebands, because their amplitudes can be influenced by the finite spectral resolution and spectral leakage. This distinction is particularly important when evaluating the closely spaced components generated at low modulation frequencies.

The normalized sideband amplitude makes it possible to determine the relative strength of frequency conversion. However, it does not represent the absolute frequency-conversion efficiency, which requires an energy-based analysis taking into account the relevant spectral components and the incident acoustic power. The analysis of the measured sideband amplitudes demonstrated that the periodic modulation of the local defect resulted in the redistribution of the transmitted acoustic spectrum among the frequency components surrounding the source frequency.

As shown in Figure 30, the increase in the amplitude of piston vibrations resulted in the expansion of the area of occurrence of Floquet harmonics, intensifying the phenomena related to the occurrence of a dynamic defect in the phononic structure.

The experimental results demonstrated the generation of additional spectral components related to the temporal modulation of the local defect in the analyzed modulation-frequency range. The analysis showed changes in the spectral distribution and apparent bandwidth of the transmission peaks for different modulation conditions. However, the experimental setup did not allow the direct verification of the effects obtained numerically for modulation frequencies from 5 to 200 Hz. The predicted effects, including the modulation of the reflected wave and the spatial distribution of Floquet harmonics at higher modulation frequencies, require further experimental investigation.

## 4. Conclusions

The influence of a dynamically modulated local defect on the propagation of mechanical waves in a quasi-one-dimensional phononic crystal was analyzed using the transfer matrix method (TMM), finite-difference time-domain (FDTD) simulations, and experimental measurements. The conducted analysis showed that the equilibrium cross-sectional area of the defect affects the transmission characteristics of the structure and the width of the fundamental band gap. The experimental transmission spectra confirmed the main features obtained from the numerical model, although differences in the resonant frequencies and transmission levels were observed.

The FDTD simulations made it possible to analyze the propagation of the mechanical wave in a finite phononic structure. The introduction of a defect in the fundamental band gap region resulted in the formation of local areas of increased wave amplitude and increased the amplitude of the transmitted wave compared to the structure without the defect. The analysis showed that, despite the finite length of the structure, components associated with the bandgap boundaries occurred in the spectrum of the transmitted wave. The conducted analysis showed that the defect affected not only the transmission characteristics but also the spatial distribution of the mechanical wave inside the phononic crystal.

FDTD simulations have shown that periodic modulation of a defect alters the propagation of mechanical waves and generates additional spectral components that satisfy the relationship $f_{k}=f_{s}+kf_{p}$, which is characteristic of frequency conversion in time-modulated systems. Within the numerically investigated range of modulation frequencies (5–200 Hz), an increase in this frequency led to stronger amplitude modulation of both transmitted and reflected waves, as well as changes in the spatial distribution of the generated Floquet harmonics. At lower modulation frequencies, the additional spectral components appeared primarily on the transmission side of the dynamic defect, whereas at higher frequencies, they were also observed on the incident side. The spatial distribution of the primary resonance—associated with the source frequency—remained virtually unchanged throughout the entire numerically investigated range. These results represent numerical predictions and have not been experimentally verified for modulation frequencies of 5 Hz or higher.

The FDTD simulations demonstrated that the piston motion amplitude, $A_{p}$, had a significant influence on the interaction between the propagating wave and the dynamic defect. Increasing this parameter resulted in stronger modulation of the transmitted wave amplitude, an increase in the local resonance amplitude on the side of the transmitted wave relative to the defect, and higher amplitudes of the generated Floquet harmonics. The conducted numerical analysis also showed that the range of the spectral response increased with increasing modulation amplitude. The obtained results indicated that the modulation amplitude provides an additional possibility for controlling the spectral characteristics and frequency conversion in the analyzed phononic structure.

The experimental analysis demonstrated the occurrence of additional spectral components for modulation frequencies of 0.25, 1.6, and 2.2 Hz. Increasing the modulation frequency resulted in a better separation of these components in the measured spectra. The observed differences between the experimental and numerical results may have been caused by uncertainties in the piston position and modulation amplitude, estimated at approximately 0.5 mm, as well as by manufacturing imperfections and dissipative effects. However, the individual influence of these factors on the obtained results has not been determined.

The conducted analysis showed that the dynamic modulation of a local defect makes it possible to control the transmission characteristics and obtain frequency conversion in finite phononic crystals. Further research should focus on the development of an actuator capable of operating at higher modulation frequencies, experimental verification of the responses predicted by the FDTD method, and determination of the influence of uncertainties in the defect parameters on the operation of the system.

## Figures and Tables

**Table 1:** Geometric and effective acoustic parameters of the meta-atoms used in the analyzed phononic crystals. The effective density and impedance are defined within the equivalent one-dimensional waveguide model and are geometry-dependent quantities rather than conventional bulk material properties.

	in,out	$A,A_{1},Y$	$B,B_{1}$	X
$\rho_{\mathrm{eff}}\left[10^{-9}\;s^{2}\right]$	10.15	15.38	30.78	5.10–30.78
$Z_{\mathrm{eff}}\;\left[10^{-6}\;m\;s\right]$	3.48	5.28	10.56	1.75–10.56
$S\;\left[10^{-3}\;m^{2}\right]$	1.19	1.81	3.62	0.6–3.62
$d\;\left[10^{-3}\;m\right]$	50.0	42.5	44.4	44.4

**Table 2:** Experimental amplitudes of the first-order Flotion frequencies for $A_{p}=0.017\;m$.

$f_{p}$ [Hz]	$p(f_{s})$ [mPa]	$p_{-1}$ [mPa]	$p_{+1}$ [mPa]	${\bar{R}}_{\mathrm{SB}}$ [%]
0.25	122.720	57.201	57.403	46.69
1.60	124.002	50.286	51.188	40.92
2.20	124.422	55.462	57.053	45.21

## Data Availability

The data that support the findings of this study are available from the corresponding author upon reasonable request.

## References

[1] Lu M.H., Feng L., Chen Y.F. (2009). Phononic crystals and acoustic metamaterials. Mater. Today.

[2] Liu J., Guo H., Wang T. (2020). A Review of Acoustic Metamaterials and Phononic Crystals. Crystals.

[3] Oudich M., Gerard N.J., Deng Y., Jing Y. (2022). Tailoring Structure-Borne Sound through Bandgap Engineering in Phononic Crystals and Metamaterials: A Comprehensive Review. Adv. Funct. Mater..

[4] Hussein M.I., Leamy M.J., Ruzzene M. (2014). Dynamics of Phononic Materials and Structures: Historical Origins, Recent Progress, and Future Outlook. Appl. Mech. Rev..

[5] Ma J. (2022). Phonon Engineering of Micro- and Nanophononic Crystals and Acoustic Metamaterials: A Review. Small Sci..

[6] Xiao Y., Wen J., Wen X. (2012). Longitudinal wave band gaps in metamaterial-based elastic rods containing multi-degree-of-freedom resonators. New J. Phys..

[7] Jia Z., Chen Y., Yang H., Wang L. (2018). Designing Phononic Crystals with Wide and Robust Band Gaps. Phys. Rev. Appl..

[8] Huang W., Xu J., Yao Y., Zhang Y., Wang X., Han P. (2025). Application of phononic crystals in modern engineering vibration and noise control, a review. Noise Vib. Worldw..

[9] Khelif A., Djafari-Rouhani B., Vasseur J.O., Deymier P.A. (2003). Transmission and dispersion relations of perfect and defect-containing waveguide structures in phononic band gap materials. Phys. Rev. B.

[10] Jo S.H., Yoon H., Shin Y.C., Youn B.D. (2022). Revealing defect-mode-enabled energy localization mechanisms of a one-dimensional phononic crystal. Int. J. Mech. Sci..

[11] Li Y., Zhou X., Bian Z., Xing Y., Song J. (2017). Thermal tuning of the interfacial adhesive layer on the band gaps in a one-dimensional phononic crystal. Compos. Struct..

[12] Aly A.H., Nagaty A., Khalifa Z. (2019). Piezoelectric Material And One-Dimensional Phononic Crystal. Surf. Rev. Lett..

[13] Wen X., Zhu X., Wu H.W., Li J. (2021). Realizing spatiotemporal effective media for acoustic metamaterials. Phys. Rev. B.

[14] Zhu X., Shen C. (2024). An acoustic metamaterial with space-time modulated density. J. Acoust. Soc. Am..

[15] Li J., Shen C., Zhu X., Xie Y., Cummer S.A. (2019). Nonreciprocal sound propagation in space-time modulated media. Phys. Rev. B.

[16] Bui H.N., Phi N.H., Alsaadi A., Lee J.W. (2022). Space–Time-Modulated Reconfigurable Metamaterial Based on a Field-Focused Cavity for Nonreciprocal Transmission Control and Frequency Conversion. ACS Appl. Mater. Interfaces.

[17] Fakheri M.H., Rajabalipanah H., Abdolali A. (2021). Spatiotemporal Binary Acoustic Metasurfaces. Phys. Rev. Appl..

[18] Yang Y., Jia H., Lu J., Yang Y., Liu T., Yang J., Liu Z. (2025). Acoustic Metasurface for Space-time Reflection Manipulation. Adv. Sci..

[19] Wen X., Zhu X., Fan A., Tam W.Y., Zhu J., Wu H.W., Lemoult F., Fink M., Li J. (2022). Unidirectional amplification with acoustic non-Hermitian space-time varying metamaterial. Commun. Phys..

[20] Fomenko S., Golub M., Zhang C., Bui T., Wang Y.S. (2014). In-plane elastic wave propagation and band-gaps in layered functionally graded phononic crystals. Int. J. Solids Struct..

[21] Sun J.H., Wu T.T. (2007). Propagation of acoustic waves in phononic-crystal plates and waveguides using a finite-difference time-domain method. Phys. Rev. B.

[22] (2024). Standard Test Method for Normal Incidence Determination of Porous Material Acoustical Properties Based on the Transfer Matrix Method.

[23] Li Z., Chen S., Wu Z., Yang L. (2020). An improved approach for normal-incidence sound transmission loss measurement using a four-microphone impedance tube. J. Vib. Control.

[24] Wright D.W. (2010). Time-Varying Phononic Crystals. Ph.D. Thesis.

